# Neuromuscular Activation of the Vastus Intermedius Muscle during Isometric Hip Flexion

**DOI:** 10.1371/journal.pone.0141146

**Published:** 2015-10-21

**Authors:** Akira Saito, Hiroshi Akima

**Affiliations:** 1 Graduate School of Education and Human Development, Nagoya University, Nagoya, Aichi, Japan; 2 Japan Society for the Promotion of Science, Chiyoda, Tokyo, Japan; 3 Research Center of Health, Physical Fitness & Sports, Nagoya University, Nagoya, Aichi, Japan; Semmelweis University, HUNGARY

## Abstract

Although activity of the rectus femoris (RF) differs from that of the other synergists in quadriceps femoris muscle group during physical activities in humans, it has been suggested that the activation pattern of the vastus intermedius (VI) is similar to that of the RF. The purpose of present study was to examine activation of the VI during isometric hip flexion. Ten healthy men performed isometric hip flexion contractions at 25%, 50%, 75%, and 100% of maximal voluntary contraction at hip joint angles of 90°, 110° and 130°. Surface electromyography (EMG) was used to record activity of the four quadriceps femoris muscles and EMG signals were root mean square processed and normalized to EMG amplitude during an isometric knee extension with maximal voluntary contraction. The normalized EMG was significantly higher for the VI than for the vastus medialis during hip flexion at 100% of maximal voluntary contraction at hip joint angles of 110° and 130° (P < 0.05). The onset of VI activation was 230–240 ms later than the onset of RF activation during hip flexion at each hip joint angle, which was significantly later than during knee extension at 100% of maximal voluntary contraction (P < 0.05). These results suggest that the VI is activated later than the RF during hip flexion. Activity of the VI during hip flexion might contribute to stabilize the knee joint as an antagonist and might help to smooth knee joint motion, such as in the transition from hip flexion to knee extension during walking, running and pedaling.

## Introduction

The quadriceps femoris (QF) plays an essential role in many human movements such as walking, running and pedaling. The QF consists of three mono-articular muscles, the vastus intermedius (VI), vastus lateralis (VL), and vastus medialis (VM), which anatomically act only as knee extensors; and the bi-articular rectus femoris (RF), which acts as a knee extensor and a hip flexor. Electromyography (EMG) studies have reported that activity of the RF differs from that of the mono-articular QF muscles during walking [1, 2], running [3, 4], cycling [5, 6], isometric hip flexion [7] and knee extension [8, 9].

Montgomery et al. [3] assessed the activation pattern of 11 thigh muscles, including the four QF synergists, during running using intramuscular EMG. They found that the activation profile of the VI was similar to that of the RF during the recovery phase of running, when the hip and knee joints were flexed. The magnitude of VI activity reached about 20% of the activity during maximal knee extension, without any apparent activation of the VL and VM muscles. Additionally, the VI activated later than the RF during the recovery phase of running. Although this finding indicates that RF and VI could be coactivated during hip flexion actions in running, it is unclear as to what action causes VI activity to occur after the RF activity during a hip flexion task.

Previous studies have described a non-uniform mono-synaptic excitatory reflex between RF and the vasti muscles in the cat limb [10, 11]. Eccles et al. [10] recorded motoneuron activity from individual QF muscles when the afferent nerve of RF was stimulated, and showed that projections from the RF to the VI were stronger than those from the RF to the VL and VM. This suggests that the excitatory afferent input from the RF onto α-motoneuron for the VI is closer than that from the RF onto α-motoneuron for the VL and VM.

Given the VI is coactivated with contraction of the RF, we hypothesize that the VI will be active during hip flexion movements. Thus, the purpose of the present study was to examine neuromuscular activation of the VI during isometric hip flexion. To record the EMG signals from the four synergist muscles in the QF, we used a surface EMG recording technique that has been developed for the VI muscle [12]. We hypothesized that VI activity would be greater than VL and VM activity during isometric hip flexion. The measurements were carried out at three different hip joint angles to evaluate the dependence of VI activity on the length of the RF muscle.

## Materials and Methods

### Participants

The experimental procedure, purposes, risks and benefits associated with the study were explained to 10 healthy men (means ± standard deviation (SD) age, 25.3 ± 6.9 years; height, 174.6 ± 6.3 cm; body mass, 67.2 ± 10.3 kg), who had no history of previous knee surgery and varied in physical activity level from untrained to regularly physical active, and who then provided written consent to participate in the present study. The Ethics Committee at the Research Center of Health, Physical Fitness & Sports, Nagoya University, approved the experimental protocol, which conformed to the ethical principles articulated in the Declaration of Helsinki.

### Experimental protocol

The participants attended our laboratory for familiarization trials at least one week before testing. Maximal voluntary contractions (MVCs) were performed during isometric knee extension at hip joint angles of 90°, 110° and 130° (where 180° is fully extended), and during isometric knee flexion at a hip joint angle of 90°. Participants then performed an MVC during isometric hip flexion at hip joint angles of 90°, 110° and 130°. After the MVC tests, the participants performed submaximal voluntary contraction tasks at three torque levels during isometric hip flexion at each hip joint angle. During the tasks, surface EMG signals from the four QF muscles and the long head of the biceps femoris (BF) were recorded.

### Knee extension and flexion task

The participants performed MVCs during isometric knee extension at hip joint angles of 90°, 110° and 130°, and during isometric knee flexion at a hip joint angle of 90° using a custom designed dynamometer (Takei Scientific Instruments Co. Ltd, Niigata, Japan) mounted onto a force transducer. The hip was strapped to the dynamometer, and the ankle was attached to a pad. The knee extension tasks at the three hip joint angles proceeded randomly. Knee extension and flexion torques were calculated as the product of the force and the distance between the lateral femoral epicondyle and the distal portion of the lower limb linked to the force transducer. The knee joint angle was flexed to 90° and assessed using a SG150 electrogoniometer (Biometrics Ltd., Gwent, UK).

During muscle contractions, torque was exerted by extending and flexing the knee. The participants attempted two MVCs for 3 s at each hip joint angle and rested for ≥ 1 min between attempts. When the generated torque reached a plateau during sustained phase, the participants were encouraged to further increase knee torque. If the generated torque differed by more than 5% value between attempts, participants performed an additional attempt.

### Hip flexion task

**Fig 1:**
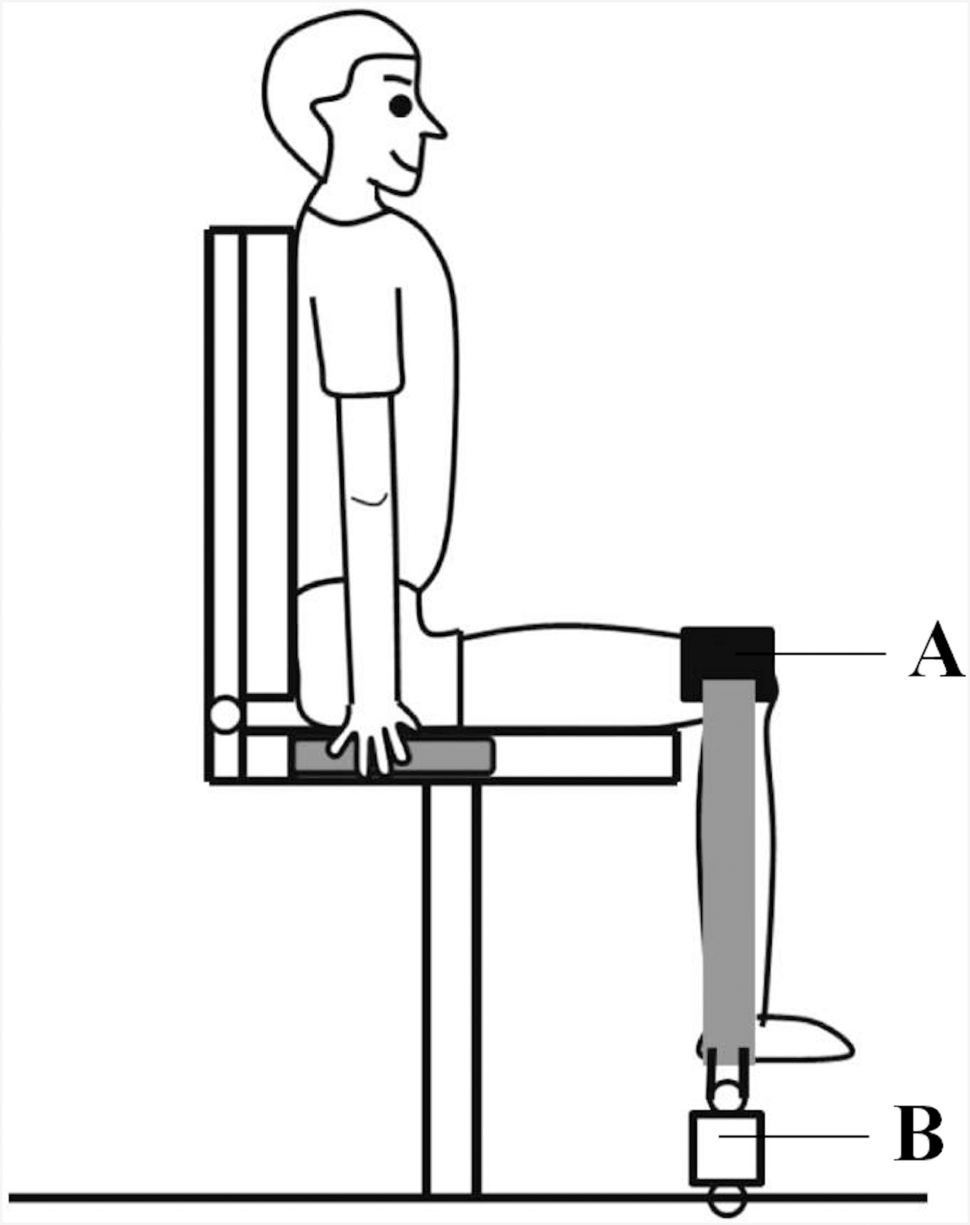
Experimental settings for measuring hip flexion torque. The pad (A) that measures the vertical force generated by hip flexion is linked to the force transducer (B).

Maximal and submaximal voluntary contractions were performed during isometric hip flexion at hip joint angles of 90°, 110° and 130° using a customized dynamometer (Takei Scientific Instruments Co. Ltd, Niigata, Japan) mounted onto a force transducer (Fig 1). The hip was strapped to the dynamometer and the distal thigh was attached to a pad. The flexion tasks at the three hip joint angles proceeded in random order. Hip flexion torque was calculated as the product of the hip flexion force and the distance between the greater trochanter and the distal portion of the thigh linked to the force transducer. The knee joint angle was flexed to 90° and assessed using the electrogoniometer.

Torque was exerted by flexing the hip. The MVC was measured as described above. Three submaximal contractions at various submaximal target torques were performed with ≥ 1 min rest between trials. The target torques were 25%, 50% and 75% of the MVC and the order of these contractions was randomized. The participants sustained hip flexion torque on a target line for 3 s. The amount of generated torque was displayed on a personal computer monitor as visual feedback with the target line for reference.

### EMG recording

Surface EMG signals were recorded from the VI, VL, VM, RF and BF muscles during the isometric contractions using active electrodes. Electrodes consisting of two silver bars (0.1 × 1 cm each with 1 cm inter-electrode distance), were used for EMG acquisition from each of these five muscles. Signals were recorded in differential amplification, with a pre-amplifier gain of 10, an input impedance of > 10^15^ Ω/0.2 pF and a 92 dB common-mode rejection ratio. The DE-2.1 sensor pre-amplifier and main amplifier, with a Bagnoli-8 bandpass filter at 20–450 Hz (Delsys, Boston, USA), were set at a gain of 10- and 100-fold, respectively, providing 1000-fold amplification. Signals were sampled at 2000 Hz (16 bits) using a Power Lab A-D converter (ADInstruments, Melbourne, Australia). EMG signals were synchronously digitized with the force and the knee angle data on a personal computer using Chart 5.5 software (ADInstruments).

Electrodes were positioned at specific locations for each muscle after shaving, abrading, and cleaning the skin with alcohol [13]. The electrodes for the VL was placed at the mid-point between the head of the greater trochanter and the inferior edge of the patella. The electrodes for the VM and RF were placed slightly proximal and medial to the patella and at the mid-point of the line joining the anterior superior iliac spine and the superior patellar pole, respectively. The electrodes for the BF were placed at the mid-point of the line joining the ischial tuberosity and the lateral femoral epicondyle. We identified the superficial regions of the VI and the BF using Logiq e ultrasound equipment (GE Healthcare, Duluth, USA). We identified the superficial region of the VI at a knee joint angle of 90° under ultrasound guidance [12, 14–19]. The VI electrodes were positioned on the skin over the superficial region of the VI. We have previously shown that there is an excellent significant correlation coefficient between surface EMG recorded from the superficial region of the VI and needle EMG recorded from the middle portion of the VI during submaximal isometric knee extension (r = 0.91–0.99) [17]. All pairs of electrodes were oriented as much as possible in parallel with the estimated direction of the muscle fascicles. A reference electrode was placed over the iliac crest.

### Data analysis

During the sustained phase, when the MVC torque was calculated, the EMG signal was sampled over 1000 ms to calculate the root mean square (RMS) amplitude during each contraction [20]. The EMG signals during the two contractions with the highest torque were used to calculate the average values of the RMS.

The RMS of the individual QF muscles during isometric hip flexion was normalized to the RMS during the isometric knee extension MVC at a hip joint angle of 90°. The RMS of the BF during isometric hip flexion was normalized to the RMS during the isometric knee flexion MVC at a hip joint angle of 90°.

**Fig 2:**
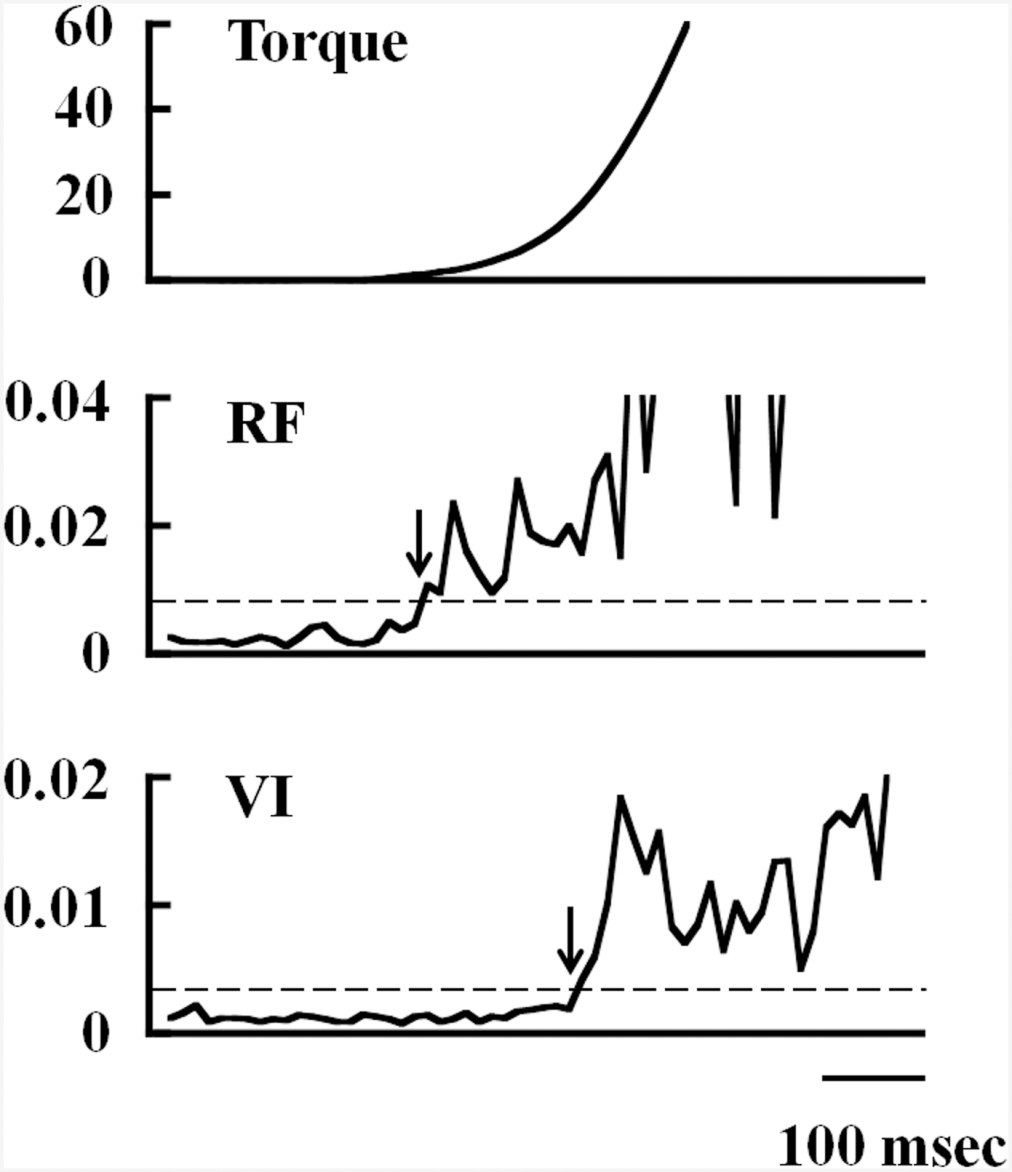
Definition of onset of muscle activation. The determined time difference between the onset of activation of rectus femoris and vastus intermedius during isometric hip flexion at maximal effort. Arrows indicate the onset of muscle activation. Horizontal dashed lines represent the threshold for determining the onset of muscle activation. RF, rectus femoris; VI, vastus intermedius.

The time difference between the onset of muscle activation of the RF and the VI during the isometric hip flexion MVC was calculated at each hip joint angle. The EMG signals during the two MVCs were RMS processed using a 10-ms moving window [21]. The onset of muscle activation for RF and VI during hip flexion was defined as the point at which the RMS amplitude reached 5% of the RMS amplitude during the isometric knee extension MVC (Fig 2). The time lag of muscle activation was averaged across the two contractions for each muscle. Onset threshold was determined as the point when EMG exceeded 5% of the RMS during knee extension, because out of thresholds of 5%, 10% and 15%, 5% resulted in an electromechanical delay for VL that was closest to previous findings [22, 23]. Electromechanical delay was calculated as the interval between the onset of EMG in the VL and the onset of torque development. The intraclass correlation coefficient between electromechanical delay in the two contractions at hip joint angles of 90°, 110° and 130° was 0.936, 0.865 and 0.716, respectively.

### Statistics

All data are expressed as mean ± SD. Isometric knee extension and hip flexion torque during MVCs at three joint angles were analyzed using a two-way (hip joint angle × movement) analysis of variance (ANOVA) with repeated measures. When a main effect was identified, Tukey’s HSD test was performed *post hoc*. Normalized EMG signals from the four QF muscles during hip flexion tasks were analyzed using a two-way (muscle × torque and hip joint angle) ANOVA with repeated measures. When a two-factor interaction was determined, Tukey’s HSD was performed *post hoc* to compare muscles at each torque level. The time lag between the onset of RF and VI muscle activation during isometric hip flexion and knee extension was analyzed using a two-way (hip joint angle × movement) ANOVA with repeated measures. When a two-factor interaction was determined, movements at each hip joint angle were compared using Student’s t-test. The level of statistical significance was set at P < 0.05. All data were statistically analyzed using IBM SPSS statistics software (version 20.0; IBM, Tokyo, Japan).

## Results

The torque exerted during the MVC significantly differed between hip flexion and knee extension (main effect of movement, P < 0.05). The isometric hip flexion MVC torque at hip joint angles of 90°, 110° and 130° was 134.8 ± 18.4, 161.0 ± 23.6 and 171.7 ± 23.8 Nm, respectively. The MVC torque at 110° and 130° was significantly higher than that at 90° (P < 0.05). The isometric knee extension MVC torque at hip joint angles of 90°, 110° and 130° was 197.3 ± 39.5, 212.9 ± 45.8 and 213.2 ± 53.9 Nm, respectively, and did not significantly differ.

**Fig 3:**
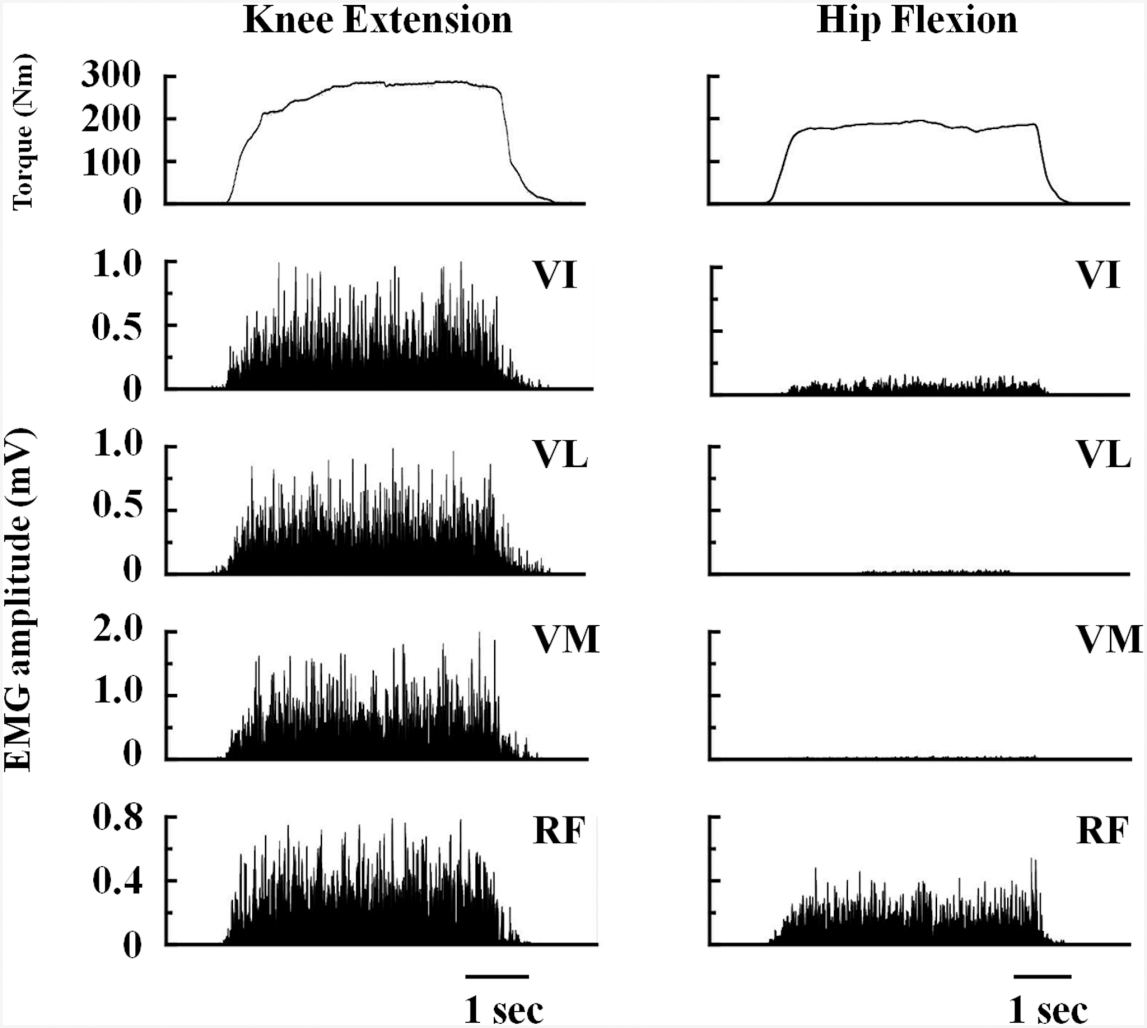
Torque and electromyographic amplitude. Rectified electromyographic signals from the four quadriceps femoris muscles and torque exerted during isometric knee extension and hip flexion with maximal effort at a hip joint angle of 130°. VI, vastus intermedius; VL, vastus lateralis; VM, vastus medialis; RF, rectus femoris.

**Fig 4:**
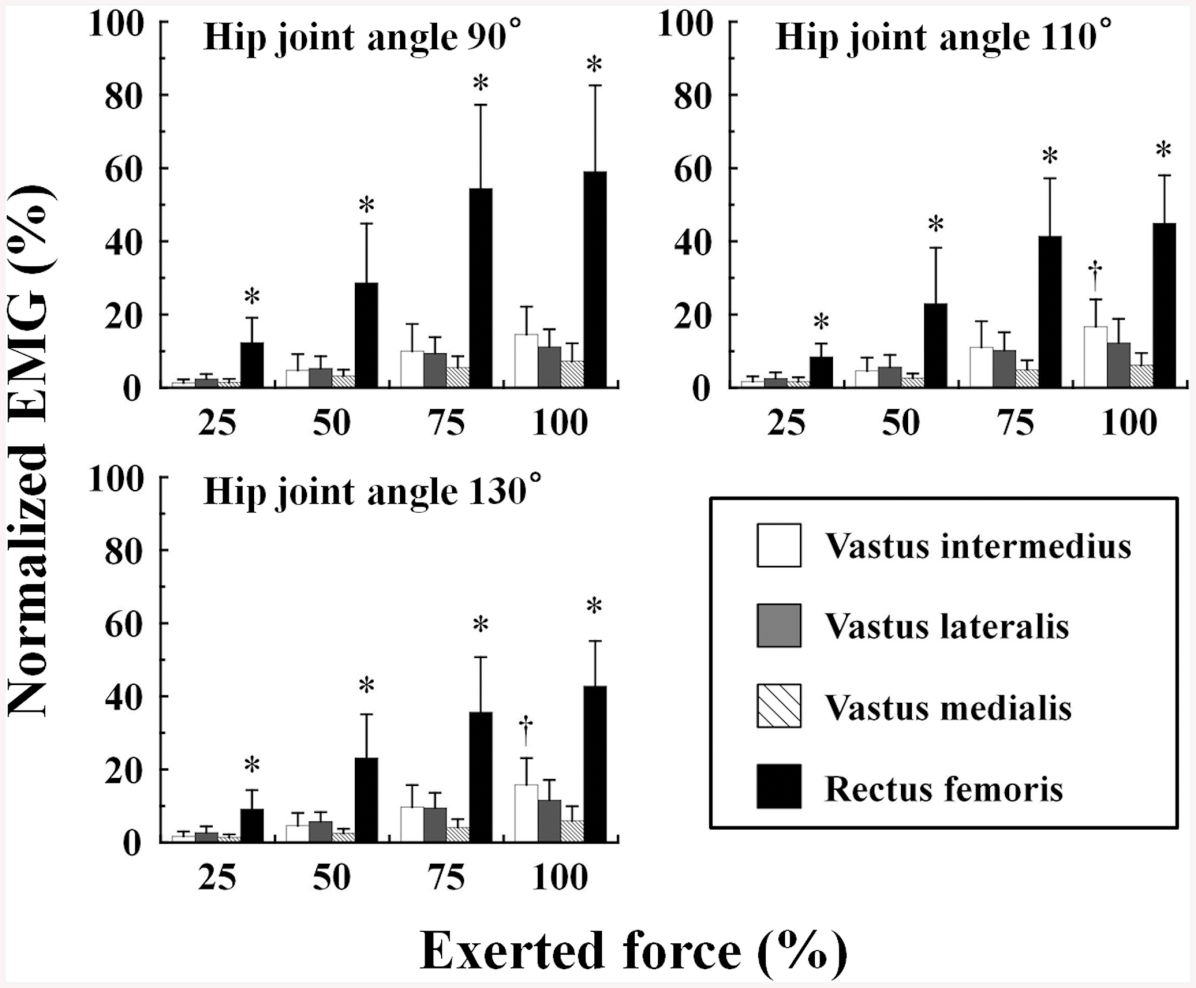
Neuromuscular activation of the four quadriceps femoris muscles at three hip joint angles. The results for each muscle at each hip joint angle were normalized to root mean square amplitude of the signal during maximal voluntary isometric contraction of the knee extensors at a hip joint angle of 90°. *, P < 0.05 vs. vastus intermedius, vastus lateralis and vastus medialis; †, P < 0.05 vs. vastus medialis.

The normalized EMG of the RF from 25% to 100% MVC was significantly higher than that of the VI, VL and VM at hip joint angles of 90°, 110° and 130°. At hip joint angles of 110° and 130°, the normalized EMG of the VI at 100% MVC was significantly higher than that of the VM (P < 0.05) (Figs 3 and 4). The normalized EMG of the QF did not significantly differ with the change in hip joint angle.

The normalized EMG of the BF was 15.6 ± 12.9%, 15.4 ± 8.2% and 20.8 ± 12.5% of MVC during isometric hip flexion at 90°, 110° and 130°, respectively, and did not significantly differ among the three hip joint angles.

**Fig 5:**
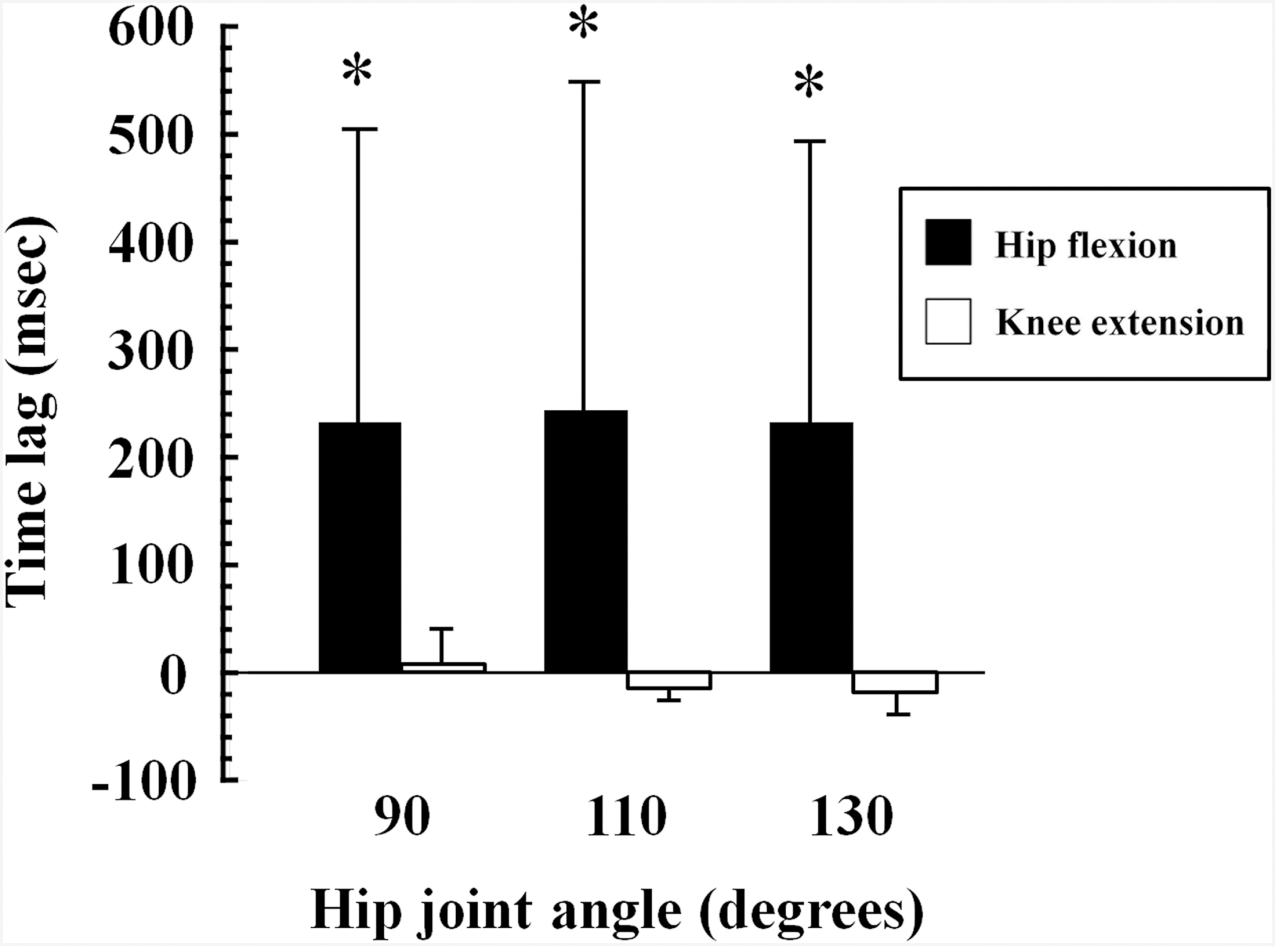
Time difference between onset of vastus intermedius and rectus femoris activation. Time lag is the duration from the onset of rectus femoris activity to the onset of vastus intermedius activity during isometric hip flexion and knee extension at maximal effort. *, P < 0.05 vs. knee extension.

The onset of VI and RF activation during the isometric hip flexion MVC was determined in 9 of the 10 participants, because VI activity for one participant during hip flexion did not reach onset threshold. During the isometric knee extension MVC, the onset of VI activation was 6.1 ± 34.7, -5.0 ± 24.6 and -19.4 ± 30.2 ms later than that of RF activation at hip joint angles of 90°, 110° and 130°, respectively. During the isometric hip flexion MVC, VI activation was delayed compared with RF activation, and the delay was 232.2 ± 273.4, 240.5 ± 312.3 and 230.5 ± 267.5 ms at hip joint angles of 90°, 110° and 130°, respectively. The delay in VI activation was significantly longer during hip flexion than during knee extension at each hip joint angle (N = 9, P < 0.05) (Fig 5).

During the isometric hip flexion MVC, the angle of the knee joint was 88.2 ± 5.1°, 91.6 ± 5.6° and 94.2 ± 7.7° at hip joint angles of 90°, 110° and 130°, respectively.

## Discussion

The purpose of the present study was to examine the neuromuscular activation of the VI during isometric hip flexion. The main findings were that the normalized EMG was significantly higher for the VI than for the VM during isometric hip flexion MVCs at hip joint angles of 110° and 130°. The difference in the time of muscle activation onset between RF and VI was significantly longer during hip flexion contractions than during knee extension contractions.

The normalized EMG of the bi-articular RF muscle was significantly higher than that of the mono-articular VI, VL and VM muscles at all hip joint angles during isometric hip flexion contractions. The normalized EMG of the RF reached 59% of MVC in the isometric knee extension contraction at a hip joint angle of 90° (Fig 4). This finding supports previous studies that showed less activity of the RF during hip flexion than during knee extension of similar intensity [7, 24].

An important finding of the present study is that, during the isometric hip flexion MVC at hip joint angles of 110° and 130°, the EMG activity of the VI was significantly higher than that of the VM (Fig 4). The higher VI activity during hip flexion may be partly explained by the results of previous studies that have used intramuscular EMG [3, 25]. VI makes a greater contribution to knee extension torque than the three superficial QF muscles (up to 50%) [25]. Zhang et al. [25] suggested that the VI may receive excitatory input onto the α-motoneuron from the central nervous system that is distinct from that received by the superficial QF. During the middle of the stance phase of running, when the hip and knee are flexed underneath the trunk, EMG activity of the VI reached approximately 20% of the activity observed in maximal knee extension, without EMG activation of VL and VM, and a similar pattern was observed in the RF [3]. This suggests that flexion of the hip joint evokes VI activity. It is not well established that the VI is activated during hip flexion, but a novel result of the present study is that activation of the VI was higher than that of the VM.

We found that the onset of VI activation was 230–240 ms after the onset of RF activation during hip flexion at each hip joint angle, and the time difference was significantly greater for hip flexion MVC than for knee extension MVC (Fig 5). These results suggest that the VI is activated later than the RF during isometric hip flexion tasks. This may be partially explained by our previous study [26], which showed that during isometric knee flexion, antagonist coactivation was higher for the VI than for the other three QF synergists. This suggests that VI acts a primary contributor to knee joint stabilization [26]. Based on this finding, VI may be activated as an antagonist to stabilize the knee after the agonist activity of the RF during isometric hip flexion.

From an anatomical viewpoint, the surface of the VI is covered by the iliotibial band, which is connected to the tensor fasciae latae muscle, a hip flexor [27]. Thus, contraction of the tensor fasciae latae muscle may influence on stability of the knee joint. Gadikota et al. [28] measured force distribution across the knee in cadavers, and found that a pulling force exerted the iliotibial band increased loading on the lateral aspect of the knee joint and decreased loading of the medical aspect. The hip flexion task in the present study may have changed the force distribution in the knee. The contraction of hip flexor muscles that cross the knee, hence there might be a need for greater knee stabilization. Since the VI may have a functional role for the knee stabilization [26], a change of force distribution in the knee during hip flexion task may be one of the factors that caused greater EMG activity in VI than in VM.

Various specialized receptors are located in the skin, fascia, tendons and muscles [29]. Afferent input from sensory receptors is one possible explanation for the later onset of VI activation relative to RF activation during the hip flexion MVC. Considering the long latency of muscle activity from the VI, afferent input from the receptors in cutaneous tissue may excite the motoneurons of the VI muscle. Gassel [30] used an ankle jerk to induce a mono-synaptic response from the triceps surae muscle following single shock stimulation delivered to the skin surface of the ipsilateral leg, and found that the reflex was facilitated after 100–300 ms. Gassel suggested that the input from the cutaneous tissue through afferent fibers facilitated the neuromuscular activity of the triceps surae [30, 31] muscles. It is suggested that afferent input from muscle spindle could be altered with latency of 100–200 ms by a stimulation of the cutaneous afferents in the hand [32] and the Ia afferents was facilitated by stimulation of cutaneous receptors in the knee [33]. Mono-synaptic responses from the RF to the VI were stronger than those to the VL and VM via Ia afferents [10, 11]. In the current study, the distal thigh was attached by a pad to the dynamometer (Fig 1); therefore, the pressure on the skin on the surface of the thigh during hip flexion might have affected receptors in the cutaneous tissue.

One potential limitation in the present study is that we did not record the force exerted at the knee joint during isometric hip flexion. Thus, during hip flexion, the VI may have been activated by knee extension or flexion movement. We previously found that, during a maximal knee flexion, antagonistic activation of the VI was 17.3% of that during a knee extension MVC at a knee joint angle of 90° and BF activity was 72% of that during a knee flexion MVC [26]. The normalized EMG for the BF in the present study was 16% of that during the knee flexion MVC. In addition, we previously showed that VI electrodes detect a negligible amount of cross-talk from the adjacent VL [12] and BF muscles [26]. In the present study, the knee joint remained in about 90° of flexion during the isometric hip flexion, suggesting that participants generated a hip flexion torque without any knee extension torque, however the results of knee angle during isometric hip flexion is not sufficient to exclude a factor that QF was activated for knee extension or flexion.

In conclusion, we investigated the neuromuscular activation of the four QF muscles during isometric hip flexion at three hip joint angles. VI activity was significantly higher than VM activity during MVCs at hip joint angles of 110° and 130°. The difference in the time of onset of RF and VI activation was significantly greater during the hip flexion MVC than during the knee extension MVC at each hip joint angles. These results suggest that VI activity is different between hip flexion and knee extension. Activity of the VI during hip flexion might contribute as an antagonist to stabilize the knee joint, and may help to smooth knee joint motion such as in the transition from hip flexion to knee extension during walking, running and pedaling. As specific muscle activities, including joint angle dependent activity and higher antagonistic coactivation for VI, have been observed from the VI muscle [18, 25, 26], it would be interesting to investigate the change of fascicle length and pennation angle in the VI using ultrasonography [34] and magnetic resonance imaging [35, 36].
